# Exploring the mechanisms underlying the therapeutic effect of *Salvia miltiorrhiza* in diabetic nephropathy using network pharmacology and molecular docking

**DOI:** 10.1042/BSR20203520

**Published:** 2021-06-16

**Authors:** Lili Zhang, Lin Han, Xinmiao Wang, Yu Wei, Jinghui Zheng, Linhua Zhao, Xiaolin Tong

**Affiliations:** 1 Department of Endocrinology, Guang’anmen Hospital, China Academy of Chinese Medical Sciences, Beijing 100053, China; 2 Graduate College, Beijing University of Traditional Chinese Medicine, Beijing 100029, China; 3 Ruikang Hospital Affiliated to Guangxi University of Traditional Chinese Medicine, Guangxi 530011, China

**Keywords:** diabetic nephropathy, molecular docking, molecular mechanism, network pharmacology, Salvia miltiorrhiza

## Abstract

The mechanisms underlying the therapeutic effect of *Salvia miltiorrhiza* (SM) on diabetic nephropathy (DN) were examined using a systematic network pharmacology approach and molecular docking. The Traditional Chinese Medicine Systems Pharmacology (TCMSP) database was used to screen active ingredients of SM. Targets were obtained using the SwissTargetPrediction and TCMSP databases. Proteins related to DN were retrieved from the GeneCards and DisGeNET databases. A protein–protein interaction (PPI) network was constructed using common SM/DN targets in the Search Tool for the Retrieval of Interacting Genes/Proteins (STRING) database. The Metascape platform was used for Gene Ontology (GO) function analysis, and the Cytoscape plug-in ClueGO was used for Kyoto Encyclopedia of Genes and Genomes (KEGG) pathway enrichment analysis. Molecular docking was performed using iGEMDOCK and AutoDock Vina software. Pymol and LigPlos were used for network mapping. Sixty-six active ingredients and 189 targets of SM were found. Sixty-four targets overlapped with DN-related proteins. The PPI network revealed that AKT serine/threonine kinase 1 (AKT1), VEGFA, interleukin 6 (IL6), TNF, mitogen-activated protein kinase 1 (MAPK1), tumor protein p53 (TP53), epidermal growth factor receptor (EGFR), signal transducer and activator of transcription 3 (STAT3), mitogen-activated protein kinase 14 (MAPK14), and JUN were the ten most relevant targets. GO and KEGG analyses revealed that the common targets of DN and SM were mainly involved in advanced glycation end-products, oxidative stress, inflammatory response, and immune regulation. Molecular docking revealed that potential DN-related targets, including tumor necrosis factor (TNF), NOS2, and AKT1, more stably bound with salvianolic acid B than with tanshinone IIA. In conclusion, the present study revealed the active components and potential molecular therapeutic mechanisms of SM in DN and provides a reference for the wide application of SM in clinically managing DN.

## Introduction

Diabetic nephropathy (DN) is a serious complication that is common in diabetic patients. As the incidence and mortality of diabetes increase yearly, the prevalence of DN rises sharply [1] and DN has become one of the leading causes of chronic renal failure [2]. DN accounts for approximately 40% of end-stage kidney disease (ESRD) cases [3]. DN starts with microalbuminuria, which progresses to macroalbuminuria and a decreased glomerular filtration rate, eventually terminating in ESRD. Histologically, glomerular basement membrane thickening and mesangial expansion are the earliest lesions observed in patients with DN [4]. These alterations are followed by nodular glomerulosclerosis and tubulointerstitial changes, including inflammatory cell infiltration, accumulation of activated myofibroblasts, and loss of capillary architecture [5]. Although multiple factors have been shown to be involved in the pathogenesis of DN, its specific molecular mechanism is complex and unclear, leading to a lack of effective therapies.

In recent years, the effects of therapeutics used in traditional Chinese medicine (TCM) in regulating blood sugar and lipid metabolism, reducing kidney damage, delaying kidney disease, and preventing glomerular sclerosis and fibrosis have gradually been uncovered. *Salvia miltiorrhiza* (SM) has a longstanding history of use for promoting blood circulation in TCM. Its main actions are reducing blood viscosity, improving hemorheological characteristics, accelerating fibrin degradation [6], antioxidant activity [7], anti-infection activity [8], and improving glucose metabolism disorders [9]. It is often used for microvascular-related diseases, such as DN and diabetic retinopathy.

Herbal remedies used in TCM act via a multitarget and multipathway intervention strategy that exerts overall regulatory and synergistic effects, which has certain advantages for DN prevention and individualized treatment. However, the mechanism of action of SM against DN is unclear.

**Figure 1:**
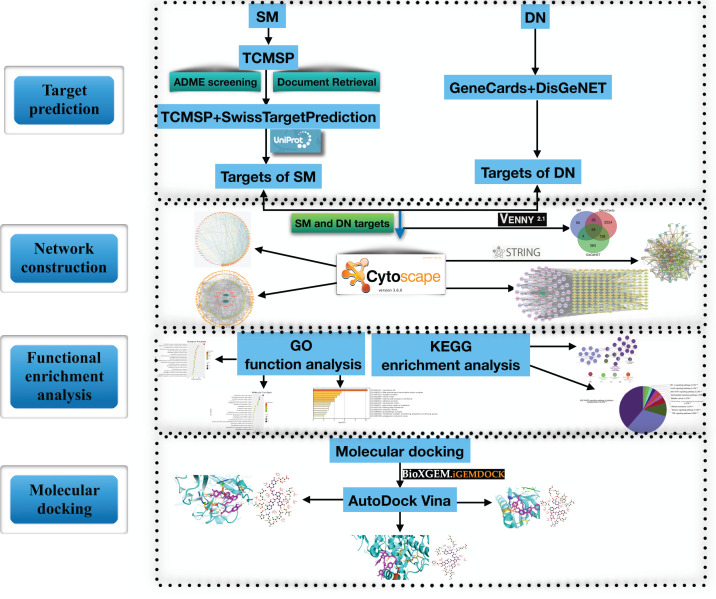
Flowchart of the network pharmacology and molecular docking study

Network pharmacology methods are effective for studying and clarifying the mechanisms underlying drug actions and include chemoinformatics, bioinformatics, network biology, and pharmacology [10,11]. The research strategy of network pharmacology is in line with the integral view on disease in TCM [12,13] and provides new ideas and methods for research on TCM [14]. In this study, we utilized a network pharmacology approach to explore the main bioactive components of SM and predict their effective molecular targets and potential mechanisms in the treatment of DN. A flowchart of the study approach is shown in Figure 1.

## Materials and methods

### Screening of active components of SM

SM active components were retrieved from the TCM Systems Pharmacology Database [TCMSP, http://tcmspw.com/tcmsp.php]. We used pharmacokinetic information retrieval filters for absorption, distribution, metabolism, and excretion (ADME) screening based on oral bioavailability (OB) ≥ 30% and drug-likeness (DL) ≥ 0.18. Active compounds without potential target information were excluded. Some active compounds that were not predicted but reported in the literature were also included.

### Construction of an active component–target network

Targets were obtained from the TCMSP and SwissTargetPrediction databases (http://www.Swisstargetprediction.ch). Then, the targets were standardized in the UniProt (https://www.uniprot.org) database with status set as ‘reviewed’ and species set as ‘human’ [15]. After removing duplicates, a database of SM compounds and their targets was constructed. Finally, a visual network was established using Cytoscape v.3.6.0 software.

### Determination of potential DN-related targets

Potential DN-related targets were retrieved from the Human Gene Database (GeneCards, https://www.genecards.org/) and the DisGeNET Database (https://www.disgenet.org/home/) using the search term ‘diabetic nephropathy.’

### Determination of DN-related targets of the active components

Screened targets of the active components and DN-related proteins were imported into a Venn diagram webtool (http://bioinformatics.psb.ugent.be/webtools/Venn/) for analysis, and common targets were identified as DN-related targets of the active components for further analysis.

### Construction of a protein–protein interaction network of DN-related targets of the active components

To study the interactions between the active components of SM and their target proteins, drug–disease intersection target genes were searched using the interaction database platform STRING v.11.0 (https://string-db.org/), and a protein–protein interaction (PPI) network was constructed. Search Tool for the Retrieval of Interacting Genes/Proteins (STRING) is a comprehensive multifunctional data platform [16,17] that aims to provide PPI evaluation and integration [18]. In our database search, the species was set to ‘*Homo sapiens*,’ the confidence score cutoff was set at 0.4, and other settings were set to default.

### Network construction and analysis

The targets of SM among DN-related proteins identified using STRING were further analyzed using the Cytoscape software v.3.6.0 to visualize and analyze the interaction network. We used the network analysis plug-in in the software to count the nodes in the network and analyze their roles in the network.

### Gene Ontology functional analysis

The Metascape platform (http://metascape.prg/gp/index.html) has a comprehensive annotation function, and gene annotation data are updated on a monthly basis [19]. SM targets regulating DN abnormalities were entered into the Metascape platform, and we analyzed their main biological processes (BPs) and performed enrichment analysis. The results were visualized using biological online tools.

### Kyoto Encyclopedia of Genes and Genomes pathway enrichment analysis

Kyoto Encyclopedia of Genes and Genomes (KEGG) pathway enrichment analysis was conducted using the Cytoscape plug-in ClueGO. The candidate DN-related genes targeted by SM were entered into the ClueGO plug-in, with *P* set to <0.01 and the κ score set to ≥ .53.

### Molecular docking

Using KEGG pathway enrichment analysis, we identified the potential DN-related genes targeted by SM active components. These targets were confirmed by molecular docking with the experimentally verified SM active components. The verified components were tanshinone IIA, which can improve kidney hypertrophy and 24-h urine protein excretion [20], and salvianolic acid B, which can inhibit the proliferation of mesangial cells and the production of extracellular matrix induced by high glucose in a dose-dependent manner [21]. Crystal structures of the verified components were obtained from the RCSB Protein Data Bank (PDB, https://www.rcsb.org/) [22]. The compound structure was saved as a docking ligand in MOL2 format. The iGEMDOCK software was used for molecular docking. The software automatically uses default parameters during standard docking.

From the molecular docking results, we selected the top five receptor proteins with the lowest energy value and the ligand that bound to these receptor proteins most stably and ran AutoDock Vina for docking. Pymol and LigPlos software was used for network visualization and construction, respectively.

## Results

### Screening of active components of SM

From the TCMSP database, we obtained 202 known active compounds of SM, which we screened based on OB ≥ 30% and DL ≥ 0.18, yielding 65 active ingredients that met the conditions. In addition, we also included the known active compound salvianolic acid B, which was screened out by ADME. Finally, 66 active components were selected for further analysis (Table 1).

**Table 1:** Basic information on the main active ingredients of SM

Mol ID	Molecule name	OB%	DL
MOL001601	1,2,5,6-tetrahydrotanshinone	38.75	0.36
MOL001659	poriferasterol	43.83	0.76
MOL001771	poriferast-5-en-3β-ol	36.91	0.75
MOL001942	isoimperatorin	45.46	0.23
MOL002222	sugiol	36.11	0.28
MOL002651	dehydrotanshinone II A	43.76	0.4
MOL002776	baicalin	40.12	0.75
MOL000569	digallate	61.85	0.26
MOL000006	luteolin	36.16	0.25
MOL007036	5,6-dihydroxy-7-isopropyl-1,1-dimethyl-2,3-dihydrophenanthren-4-one	33.77	0.29
MOL007041	2-isopropyl-8-methylphenanthrene-3,4-dione	40.86	0.23
MOL007045	3α-hydroxytanshinoneIIa	44.93	0.44
MOL007048	(E)-3-[2-(3,4-dihydroxyphenyl)-7-hydroxy-benzofuran-4-yl]acrylic acid	48.24	0.31
MOL007049	4-methylenemiltirone	34.35	0.23
MOL007050	2-(4-hydroxy-3-methoxyphenyl)-5-(3-hydroxypropyl)-7-methoxy- 3-benzofurancarboxaldehyde	62.78	0.4
MOL007058	formyltanshinone	73.44	0.42
MOL007059	3-β-hydroxymethyllenetanshiquinone	32.16	0.41
MOL007061	methylenetanshinquinone	37.07	0.36
MOL007063	przewalskin a	37.11	0.65
MOL007064	przewalskin b	110.32	0.44
MOL007068	przewaquinone B	62.24	0.41
MOL007069	przewaquinone c	55.74	0.4
MOL007070	(6S,7R)-6,7-dihydroxy-1,6-dimethyl-8,9-dihydro-7H-naphtho[8,7-g]benzofuran-10,11-dione	41.31	0.45
MOL007071	przewaquinone f	40.31	0.46
MOL007077	sclareol	43.67	0.21
MOL007079	tanshinaldehyde	52.47	0.45
MOL007081	danshenol B	57.95	0.56
MOL007082	danshenol A	56.97	0.52
MOL007085	salvilenone	30.38	0.38
MOL007088	cryptotanshinone	52.34	0.4
MOL007093	dan-shexinkum d	38.88	0.55
MOL007094	danshenspiroketallactone	50.43	0.31
MOL007098	deoxyneocryptotanshinone	49.4	0.29
MOL007100	dihydrotanshinlactone	38.68	0.32
MOL007101	dihydrotanshinone I	45.04	0.36
MOL007105	epidanshenspiroketallactone	68.27	0.31
MOL007107	C09092	36.07	0.25
MOL007108	isocryptotanshi-none	54.98	0.39
MOL007111	isotanshinone II	49.92	0.4
MOL007115	manool	45.04	0.2
MOL007119	miltionone I	49.68	0.32
MOL007120	miltionone II	71.03	0.44
MOL007121	miltipolone	36.56	0.37
MOL007122	miltirone	38.76	0.25
MOL007124	neocryptotanshinone ii	39.46	0.23
MOL007125	neocryptotanshinone	52.49	0.32
MOL007127	1-methyl-8,9-dihydro-7H-naphtho[5,6-g]benzofuran-6,10,11-trione	34.72	0.37
MOL007130	prolithospermic acid	64.37	0.31
MOL007132	(2R)-3-(3,4-dihydroxyphenyl)-2-[(Z)-3-(3,4-dihydroxyphenyl)acryloyl]oxy-propionic acid	109.38	0.35
MOL007141	salvianolic acid g	45.56	0.61
MOL007142	salvianolic acid j	43.38	0.72
MOL007143	salvilenone I	32.43	0.23
MOL007145	salviolone	31.72	0.24
MOL007150	(6S)-6-hydroxy-1-methyl-6-methylol-8,9-dihydro-7H-naphtho[8,7-g] benzofuran-10,11-quinone	75.39	0.46
MOL007151	tanshindiol B	42.67	0.45
MOL007152	przewaquinone E	42.85	0.45
MOL007154	tanshinone iia	49.89	0.4
MOL007155	(6S)-6-(hydroxymethyl)-1,6-dimethyl-8,9-dihydro-7H-naphtho[8,7-g]benzofuran-10,11-dione	65.26	0.45
MOL007156	tanshinone VI	45.64	0.3
MOL006824	α-amyrin	39.51	0.76
MOL007118	microstegiol	39.61	0.28
MOL007123	miltirone II	44.95	0.24
MOL007149	NSC 122421	34.49	0.28
MOL007140	(Z)-3-[2-[(E)-2-(3,4-dihydroxyphenyl)vinyl]-3,4-dihydroxy-phenyl]acrylic acid	88.54	0.26
MOL007051	6-o-syringyl-8-o-acetyl shanzhiside methyl ester	46.69	0.71
MOL007074	salvianolic acid b	3.01	0.41

### Determination of targets of SM active components and active component–target network construction

**Figure 2:**
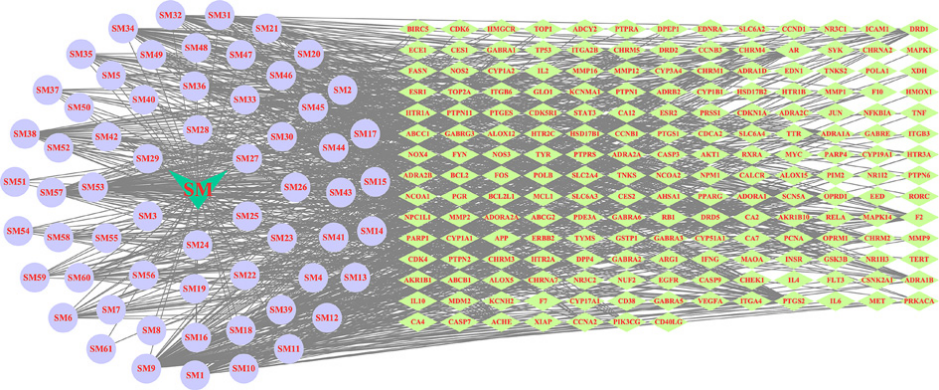
‘Ingredients–targets’ network construction The light cyan prism nodes represent the targets, the light purple round nodes represent SM ingredients.

Candidate targets of active components in SM were searched from the TCMSP and SwissTargetPrediction databases. After UniProt standardization, deduplication occurred. After the removal of duplicate data, 189 targets were identified (Supplementary Table S1). Next, we used CytoScape 3.6.0 software to build a ‘active ingredients–targets’ interaction network, as shown in Figure 2.

### Determination of DN-related targets

We retrieved 1189 and 3084 DN-related disease targets obtained from the GeneCards and DisGeNET databases, respectively.

### Drug–disease intersection targets

**Figure 3:**
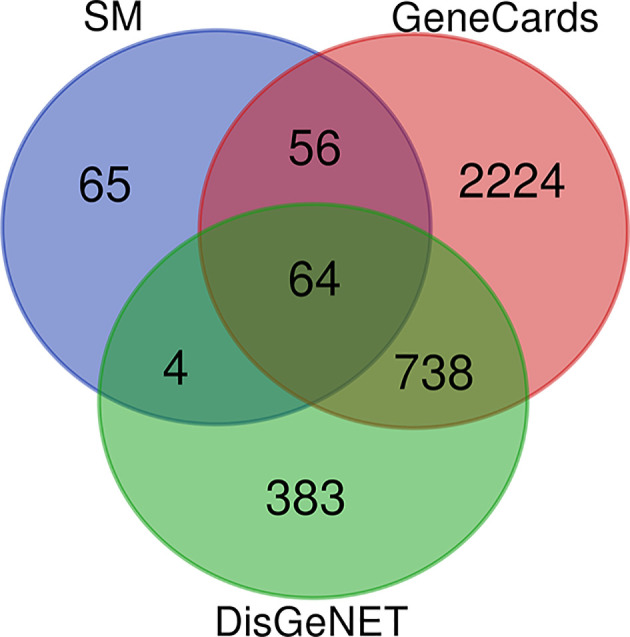
SM/DN common target genes

Venn analysis was performed using the 189 targets of SM active components and 1189 and 3084 DN-related target genes, and 64 drug–disease intersection gene targets were obtained for further analysis, as shown in Figure 3 and Table 2. Information on these targets is provided in Supplementary Table S2.

**Table 2:** Potential targets of SM against DN

Serial number	Protein name	Gene name	UniProt ID
1	peroxisome proliferator activated receptor γ	PPARG	P37231
2	vascular endothelial growth factor A	VEGFA	P15692
3	insulin receptor	INSR	P06213
4	interleukin 6	IL6	P05231
5	nitric oxide synthase 3	NOS3	P29474
6	tumor necrosis factor	TNF	P01375
7	solute carrier family 2 member 4	SLC2A4	P14672
8	AKT serine/threonine kinase 1	AKT1	P31749
9	signal transducer and activator of transcription 3	STAT3	P40763
10	dipeptidyl peptidase 4	DPP4	P27487
11	intercellular adhesion molecule 1	ICAM1	P05362
12	tumor protein p53	TP53	P04637
13	interleukin 10	IL10	P22301
14	endothelin 1	EDN1	P05305
15	CD40 ligand	CD40LG	P29965
16	matrix metallopeptidase 9	MMP9	P14780
17	interleukin 4	IL4	P05112
18	nitric oxide synthase 2	NOS2	P35228
19	interferon gamma	IFNG	P01579
20	interleukin 2	IL2	P60568
21	heme oxygenase 1	HMOX1	P09601
22	matrix metallopeptidase 2	MMP2	P08253
23	mitogen-activated protein kinase 1	MAPK1	P28482
24	prostaglandin-endoperoxide synthase 2	PTGS2	P35354
25	mitogen-activated protein kinase 14	MAPK14	Q16539
26	nuclear receptor subfamily 3 group C member 2	NR3C2	P08235
27	caspase 3	CASP3	P42574
28	Jun proto-oncogene, AP-1 transcription factor subunit	JUN	P05412
29	xanthine dehydrogenase	XDH	P47989
30	estrogen receptor 1	ESR1	P03372
31	matrix metallopeptidase 1	MMP1	P03956
32	phosphatidylinositol-4,5-bisphosphate 3-kinase catalytic subunit gamma	PIK3CG	P48736
33	endothelin receptor type A	EDNRA	P25101
34	epidermal growth factor receptor	EGFR	P00533
35	serine protease 1	PRSS1	P07477
36	RELA proto-oncogene, NF-kB subunit	RELA	Q04206
37	caspase 9	CASP9	P55211
38	prostaglandin-endoperoxide synthase 1	PTGS1	P23219
39	5-hydroxytryptamine receptor 2A	HTR2A	P28223
40	fatty acid synthase	FASN	P49327
41	cyclin dependent kinase inhibitor 1A	CDKN1A	P38936
42	coagulation factor X	F10	P00742
43	glutathione S-transferase pi 1	GSTP1	P09211
44	BCL2 apoptosis regulator	BCL2	P10415
45	integrin subunit beta 3	ITGB3	P05106
46	protein tyrosine phosphatase non-receptor type 2	PTPN2	P17706
47	MDM2 proto-oncogene	MDM2	Q00987
48	integrin subunit alpha 2b	ITGA2B	P08514
49	matrix metallopeptidase 12	MMP12	P39900
50	nuclear receptor subfamily 1 group I member 2	NR1I2	O75469
51	caspase 7	CASP7	P55210
52	lymphocyte differentiation antigen CD38	CD38	P28907
53	Glyoxalase I	GLO1	Q04760
54	arachidonate 12-lipoxygenase	ALOX12	P18054
55	aldose reductase (by homology)	AKR1B1	P15121
56	protein-tyrosine phosphatase 1C	PTPN6	P29350
57	LXR-α	NR1H3	Q13133
58	protein-tyrosine phosphatase 2C	PTPN11	Q06124
59	arginase-1(by homology)	ARG1	P05089
60	poly[ADP-ribose] polymerase-1	PARP1	P09874
61	adenosine A1 receptor (by homology)	ADORA1	P30542
62	NADPH oxidase 4	NOX4	Q9NPH5
63	tyrosine-protein kinase SYK	SYK	P43405
64	cytochrome P450 19A1	CYP19A1	P11511

### PPI network analysis

**Figure 4:**
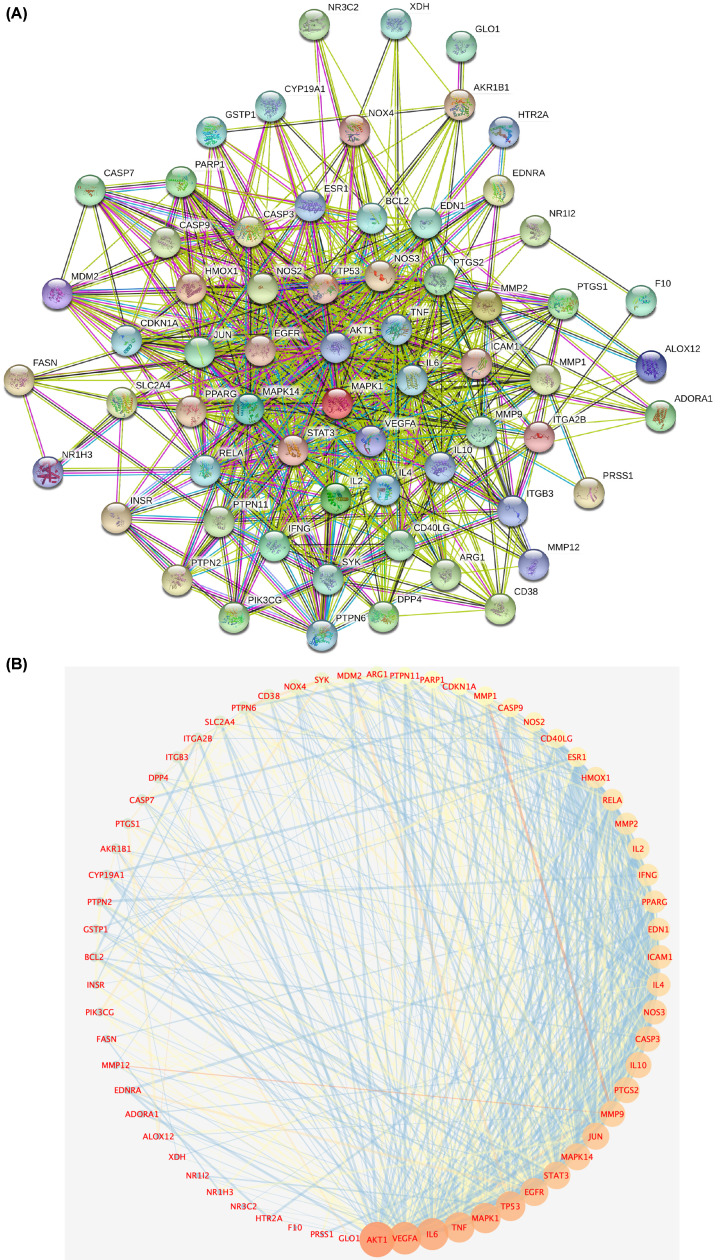
PPI network analysis (**A**) PPI network of targets generated using STRING 11.0. Nodes represent proteins. Edges represent PPIs. (**B**) Potential targets are arranged counterclockwise according to the degree value from large to small.

The 64 drug–disease intersection gene targets were analyzed using a PPI network constructed using the STRING database, as shown in Figure 4A. The network 64 nodes and 704 edges, and the average node degree was 21.3, with a PPI enrichment *P*-value of <1.0e-16 (Figure 4A). The results of STRING analysis were imported into Cytoscape software. The network analysis plug-in was used to count the nodes in the network graph and analyze their connectivity according to the node degree; the greater the node degree, the more biological functions the node has in the network. The network was constructed as shown in Figure 4B. The ten most-connected targets were AKT serine/threonine kinase 1 (AKT1), vascular endothelial growth factor A (VEGFA), interleukin 6 (IL6), tumor necrosis factor (TNF), mitogen-activated protein kinase 1 (MAPK1), tumor protein p53 (TP53), epidermal growth factor receptor (EGFR), signal transducer and activator of transcription 3 (STAT3), mitogen-activated protein kinase 14 (MAPK14), and transcription factor AP-1 (JUN), indicating their significance in the network (Figure 4B).

### Gene Ontology functional analysis

**Figure 5:**
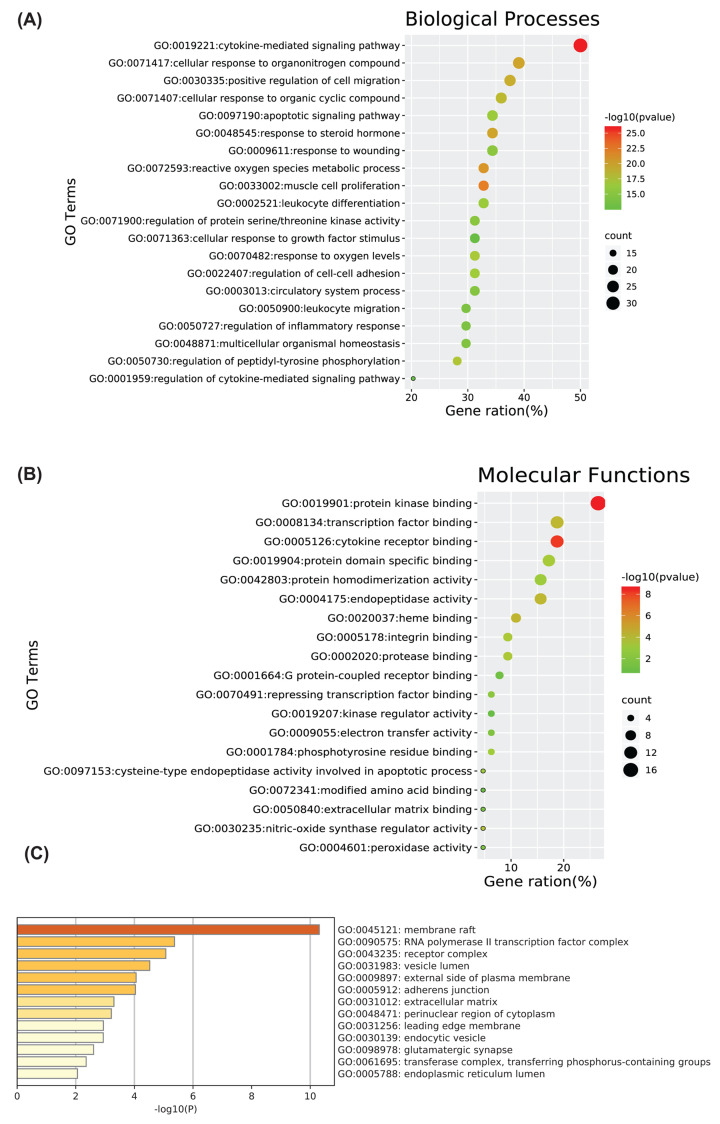
GO enrichment analysis Included are (**A**) BP terms, (**B**) molecular function (MF) terms, and (**C**) cellular component (CC) terms. (A,B) Node color is displayed in a gradient from red to green in descending order of the *P*-value. The size of the nodes is arranged in ascending order of the number of genes. (C) Sorted by the importance of –log10(P) of each lane.

The Metascape data platform was used for enrichment analysis of the 64 relevant DN-related targets of SM, and the results were visualized using biological online tools. A total of 1557 BP Gene Ontology (GO) terms were enriched, and the 20 most significantly enriched BP terms (*P*<0.01) were selected for analysis. The results showed that BPs enriched in DN-related SM targets mainly included cytokine-mediated signaling pathway, apoptotic signaling pathway, positive regulation of cell migration, reactive oxygen species metabolic process, regulation of inflammatory response, regulation of cell–cell adhesion, response to oxygen levels, cellular response to growth factor stimulus, and regulation of protein serine/threonine kinase activity (Figure 5A).

A total of 90 molecular function (MF) GO terms were enriched, and the 19 most significantly enriched MF terms based on *P*<0.01 were selected for analysis. The results showed that the intersection genes were mainly enriched in protein kinase binding, transcription factor binding, cytokine receptor binding, integrin binding, cysteine-type endopeptidase activity involved in apoptotic process, kinase regulator activity, heme binding, protein domain-specific binding, endopeptidase activity, nitric-oxide synthase regulator activity, and many other MFs related to the above genes (Figure 5B).

A total of 38 cellular component (CC) GO terms were enriched, and the 13 most significantly enriched CC terms based on *P*<0.01 were selected for analysis. The results showed that the intersection genes were mainly enriched in membrane rafts, RNA polymerase II transcription factor complex, external side of plasma membrane, extracellular matrix, adherens junction, and glutamatergic synapse (Figure 5C). Detailed node attribute information of the GO analysis results is provided in Supplementary Table S3.

### KEGG pathway enrichment analysis

**Figure 6:**
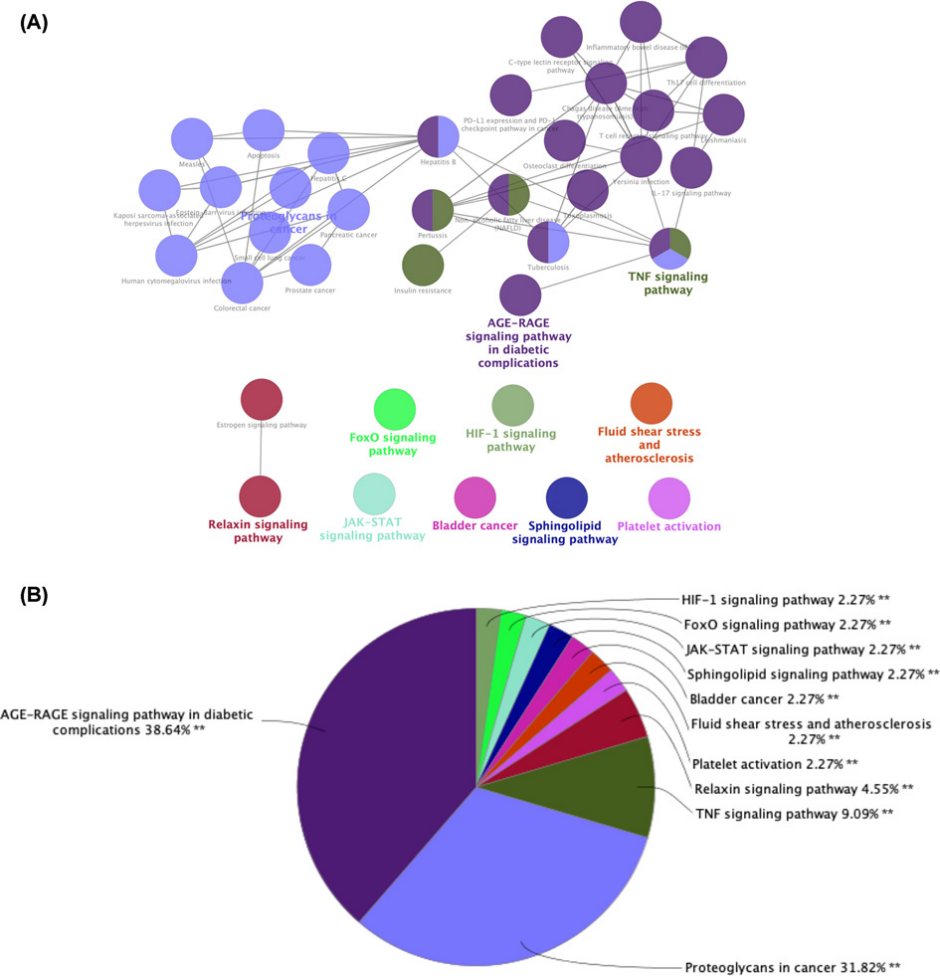
KEGG pathway analysis of potential targets of SM among DN-related proteins using the ClueGO plug-in (**A**) The KEGG term is indicated as a node, and the size of the node indicates its importance. Only the most significant terms in the group are labeled. (**B**) Pie chart presenting the percentage of genes involved in different biological functions and signaling pathways in the total number of genes that are intersected.

To reveal the potential mechanism underlying the therapeutic effect of SM in DN, we performed KEGG pathway enrichment analysis of the 64 intersection gene targets using the Cytoscape plug-in ClueGO. The screen was based on *P*<0.01 and a κ score ≥ 0.53 in order to visualize the results of KEGG enrichment (Figure 6A), and we used a pie chart to describe the percentages of genes involved in the different biological functions and signal pathways among the total number of intersection genes (Figure 6B). The results showed that 38 terms were enriched, including the AGE-RAGE signaling pathway in diabetic complications, TNF signaling pathway, JAK-STAT signaling pathway, FoxO signaling pathway, and HIF-1 signaling pathway. In addition, we found some other pathways, including fluid shear stress and atherosclerosis, platelet activation, and relaxin signaling pathway. These results revealed that SM alleviated DN by improving human immunity, anti-inflammatory action, reducing levels of advanced glycation end-products, antioxidant stress response, and regulating other pathways in response to harmful alien organisms. Detailed node attribute information of the KEGG analysis results is provided in Supplementary Table S4.

### Molecular docking study

According to the results of KEGG pathway enrichment analysis, we selected the AGE-RAGE signaling pathway in diabetic complications, which had the largest percentage of genes involved in different biological functions and signaling pathways among the total number of intersection genes, for further analysis. Based on drug–target correspondence, the target proteins in this pathway were molecularly docked. The 16 target proteins enriched in the AGE-RAGE signaling pathway in diabetic complications were AKT1, BCL2, CASP3, EDN1, ICAM1, IL6, JUN, MAPK1, MAPK14, MMP2, NOS3, NOS2, RELA, STAT3, TNF, and VEGFA. We selected the experimentally verified SM active molecules tanshinone IIA and salvianolic acid B and molecularly docked the 16 target proteins with them. The most stable conformation is the one with the lowest binding energy. The 16 potential DN-related targets more stably bound to salvianolic acid B than to tanshinone IIA (Table 3).

**Table 3:** Docking scores of targets with tanshinone IIA and salvianolic acid B (kcal.mol^–1^)

Target name	PDBID	Tanshinone IIA	Salvianolic acid B	Canagliflozin
MMP2	1EAK	−88.21	−127.25	−109.23
EDN1	1EDP	−62	−110.92	−92.83
RELA	1NFI	−81.98	−151.32	−92.46
NOS3	1NIW	−92.71	−131.05	−113.3
JUN	1S9K	−87.31	−147.78	−99.95
AKT1	1UNQ	−79.22	−148.03	−102.74
BCL2	1YSW	−78.34	−129.71	−113.85
TNF	2E7A	−90.62	−146.4	−113.05
MAPK14	2NPQ	−84.34	−139.24	−102.14
BCL2	2O2F	−82.16	−133.24	−99.02
BCL2	2O21	−89.69	−136.58	−105.02
BCL2	2O22	−97.25	−135.9	−104.91
NOS2	3E7G	−89.58	−162.49	−109.28
CASP3	3KJF	−81.44	−130.26	−91.52
VEGFA	3V2A	−76.85	−121.29	−91.19
IL6	4CNI	−89.55	−125.52	−98.18
MAPK1	4IZ5	−78.5	−144.95	−97.71
STAT3	4ZIA	−84.1	−136.45	−103.68
ICAM1	5MZA	−80.39	−125.56	−98.36

**Figure 7:**
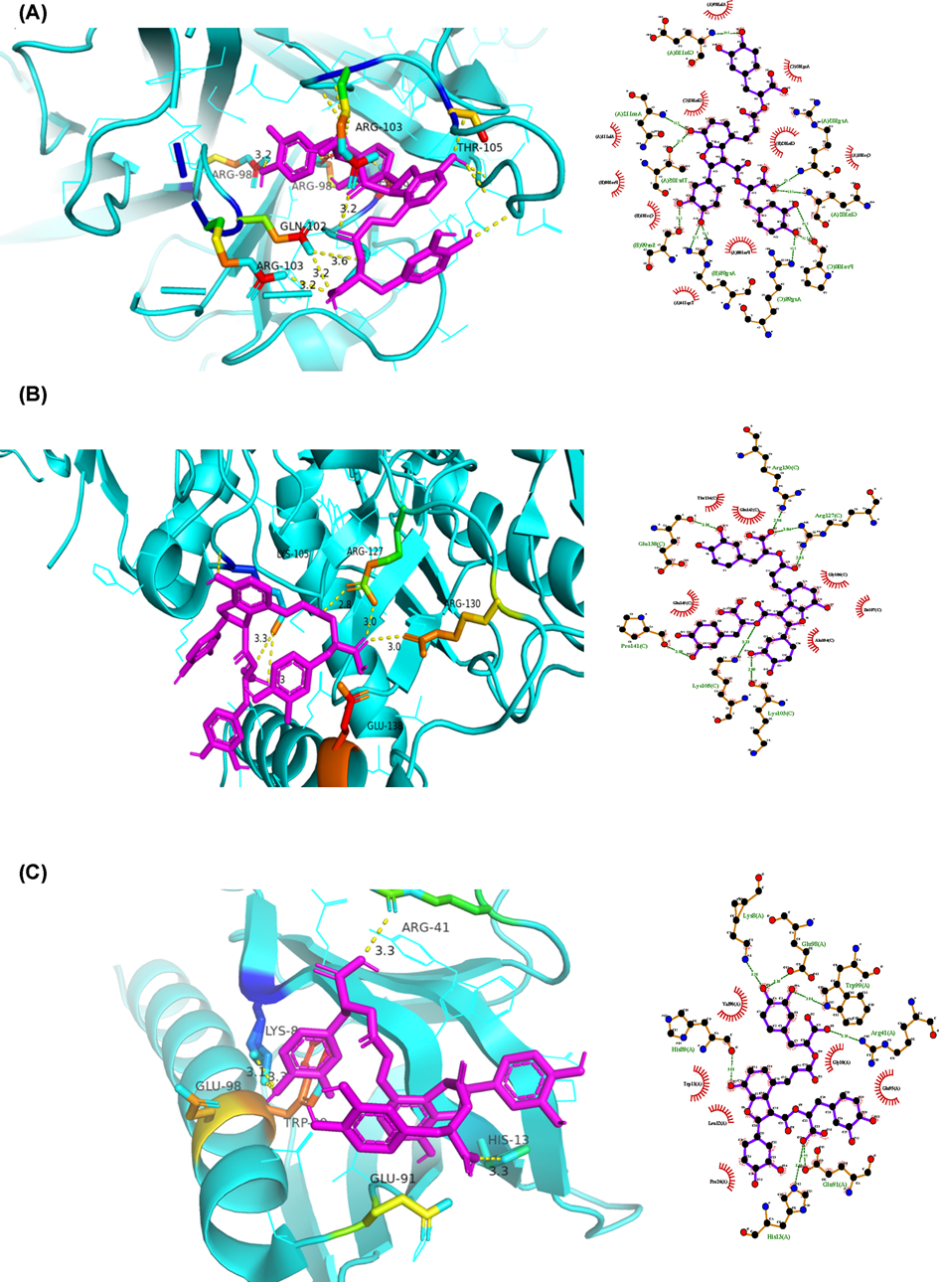
Molecular docking Molecular models of the binding of salvianolic acid B with (**A**) TNF, (**B**) NOS2, and (**C**) AKT1 shown as 3D and 2D diagrams.

Using AutoDock Vina software, the five target proteins with the lowest energy value in the molecular docking (AKT1, NOS2, TNF, JUN, and RELA) were molecularly docked with the active component salvianolic acid B. Figure 7 shows the best docking combinations for the target proteins and salvianolic acid B, including TNF, NOS2, and AKT1, with binding energies of –9.3, –6.6, and –6.4 kcal/mol, respectively. This shows that salvianolic acid B has a good binding ability to these targets.

## Discussion

The mechanisms of action of TCM therapeutics are complex, with multiple components and targets. When the pathogenesis of disease has not been clarified, it becomes more difficult to analyze the mechanism of action of a TCM therapeutic. Network pharmacology combines system network analysis and pharmacology. It allows systematic study of the effective components, targets, and pathways of drugs at the molecular level, improving our understanding of the interactions between components, targets, and pathways.

In the present study, TCM active component–target network analysis revealed that luteolin, tanshinone IIA, salviolone, salvianolic acid B, dihydrotanshinlactone, and other active ingredients can act on multiple targets in the network. This finding suggests that these components may be important for the therapeutic effect of SM in DN and warrant further exploration. Luteolin has the most potential targets, followed by tanshinone IIA. According to previous reports, luteolin not only increases insulin-mediated glucose uptake and enhances insulin sensitivity [23] but also inhibits high glucose-induced vascular endothelial growth factor (VEGF) [24], reducing reactive oxygen species generation and lipid accumulation [25]. This shows that luteolin can improve insulin resistance and regulate glucose and lipid metabolism. Tanshinone IIA reduces vascular intimal hyperplasia, improves tissue blood perfusion, improves kidney microcirculation, removes intracellular oxygen free radicals, improves blood lipids, promotes anticoagulation, and exerts various other actions [26], such as anti-inflammatory and antioxidant actions [27,28], thereby reducing kidney damage. Salvianolic acid B is a water-soluble compound with the highest activity and content in SM [29]. A number of basic studies have shown that salvianolic acid B has potential therapeutic effects on renal microcirculation. Salvianolic acid B has anti-oxidation [30], anti-inflammatory [31], neuroprotection [32], and anti-fibrosis [33] effects. Salvianolic acid B can be delivered to the kidneys to reduce the progression of renal fibrosis [34], thereby protecting renal function and delaying DN progression.

In the PPI network of 64 targets of SM acting in DN, AKT1, VEGFA, IL6, TNF, MAPK1, TP53, EGFR, STAT3, MAPK14, and JUN were the top ten targets based on node degree. These proteins are regarded core proteins and may play important roles in the therapeutic effect of SM in DN. These proteins are involved in oxidative stress, inflammation, vascular permeability, and immune regulation. For example, the activation of AKT1 promotes cell proliferation and inhibits cell apoptosis. It is an important player in the immune inflammatory mechanism of DN [35]. It is closely related to mesangial matrix proliferation, basement membrane thickening, podocyte damage, and renal tubular epithelial cell transdifferentiation [36]. IL6 and TNF have immunomodulatory and pro-inflammatory effects [37]. VEGFA is related to vascular permeability in patients with DN [38]. Activated eGFR up-regulates reactive oxygen species production and endoplasmic reticulum stress, and this mechanism plays an important role in DN onset [39]. Therefore, it can be inferred that luteolin, tanshinone IIA, and salvianolic acid B, the main active components of SM, reduce oxidative stress and inhibit the expression of inflammatory mediators, such as IL-10, IL-6, and TNF, thus delaying DN progression.

To predict the mechanism underlying the therapeutic effect of SM in DN, we performed GO enrichment analysis of the 64 potential targets. As shown in Figure 5A, the 20 most significantly enriched BP terms were mainly related to cytokines, apoptosis, reactive oxygen species, and inflammation regulation. Relevant studies have shown that DN onset and development are related to cell dysfunction and damage [40,41], chronic inflammatory infiltration [42], cell apoptosis, and oxidative stress [43]. This indicates that the main targets are important for multiple BPs. MFs enriched in targets mainly included cytokine receptor binding, integrin binding, endopeptidase activity, transcription factor binding, protein kinase binding, and heme binding (Figure 5B). The targets involved mainly included VEGFA, PTGS2, DDP4, TNF, and NOS2, which are mainly involved in oxidative stress, the inflammatory response, and immune regulation. DDP4 inhibitors are of great significance for reducing blood sugar levels in diabetic patients and delaying the onset and development of DN [44,45]. In addition, as shown in Figure 5C, CCs enriched in targets mainly included membrane raft, RNA polymerase II transcription factor complex, external side of plasma membrane, extracellular matrix, and adherens junction. These enriched functions involved top targets, such as TNF and JUN. Together, these findings illustrate the complexity of the pathological mechanism of DN.

To further explore the potential mechanism of SM in treating DN, we conducted KEGG analysis of the 64 potential targets of SM acting in DN. As shown in Figure 6, the pathways related to DN, including the AGE-RAGE signaling pathway in diabetic complications, TNF signaling pathway, JAK-STAT signaling pathway, and FoxO signaling pathway, mainly involve three aspects: (1) accumulation of advanced glycation end-products: normally, the glycation reaction proceeds very slowly. However, the response is obviously accelerated in the hyperglycemic state, and the aggregation of AGEs in tissues and their binding with RAGE, a specific receptor, produces cytotoxic effects and damages the kidneys, which may be the key factors contributing to DN. Studies have shown that the AGE/RAGE signaling pathway can promote the expression of NF-κB [46], up-regulate TGF-β1, VEGF [47], activate NADPH oxidases, induce the expression and release of inflammatory factors and adhesion factors, increase vascular permeability, increase the expression of connective tissue growth factor, and enhance oxidative stress, thus increasing proteinuria, promoting renal fibrosis, leading to DN onset and development. Studies have shown that the interaction between AGEs and RAGE leads to vasoconstriction and a procoagulant state [48], accelerates renal vascular aging and injury [49], and further promotes DN progression. (2) Immune inflammation regulation: TNF has immunomodulatory and pro-inflammatory effects [37]. TNF-α stimulates the aggregation and adhesion of inflammatory cells, increases the permeability of microvessels, and impairs glomeruli through an inflammatory response [50]. Some studies have confirmed that TNF-α levels are significantly increased in DN patients and positively correlate with the course of disease [51,52]. In addition, studies have shown that JAK/STAT signaling activation can cause immune inflammation in the kidneys [53,54] and mediates mesangial proliferation and renal tissue fibrosis associated with DN [55]. (3) Oxidative stress: FoxO mainly regulates oxidative stress, apoptosis, and immune responses through the transcription and transmission of various growth factors and cytokine signals, among which FoxO1 plays an important role in the pathogenesis of kidney disease [56]. FoxO1 activation can inhibit podocyte epithelial–mesenchymal cell transformation induced by high glucose and improve proteinuria and renal damage in diabetic mice [57]. In addition, we found other pathways, such as proteoglycans in cancer, fluid shear stress, and atherosclerosis, indicating that SM has potential applications in tumors, atherosclerosis, and other diseases. Based on the aforementioned multiple pathways, it is speculated that SM delays the progression of DN and protects renal function by participating in advanced glycation end products, oxidative stress, inflammatory response, immune regulation, and other processes.

To further explore the potential molecular mechanism of SM in the treatment of DN, we conducted molecular docking studies of 16 targets closely related to DN according to a KEGG-based screening, using the experimentally validated key components tanshinone IIA and salvianolic acid B as ligands. Results showed that the 16 potential targets had a good binding ability to salvianolic acid B, and the interactions were more stable than those with tanshinone IIA.

The present study had some limitations. We only explored the effect of SM in DN at the network pharmacology level. However, current network information technology is not comprehensive, and the accuracy of database data and real-time updates needs to be improved. Therefore, the results obtained in the present study require verification in terms of pharmacodynamics, and mechanistic experiments are needed to explain the complex multitarget, multipathway, and synergistic interactions involved in the therapeutic effects of SM.

## Conclusions

**Figure 8:**
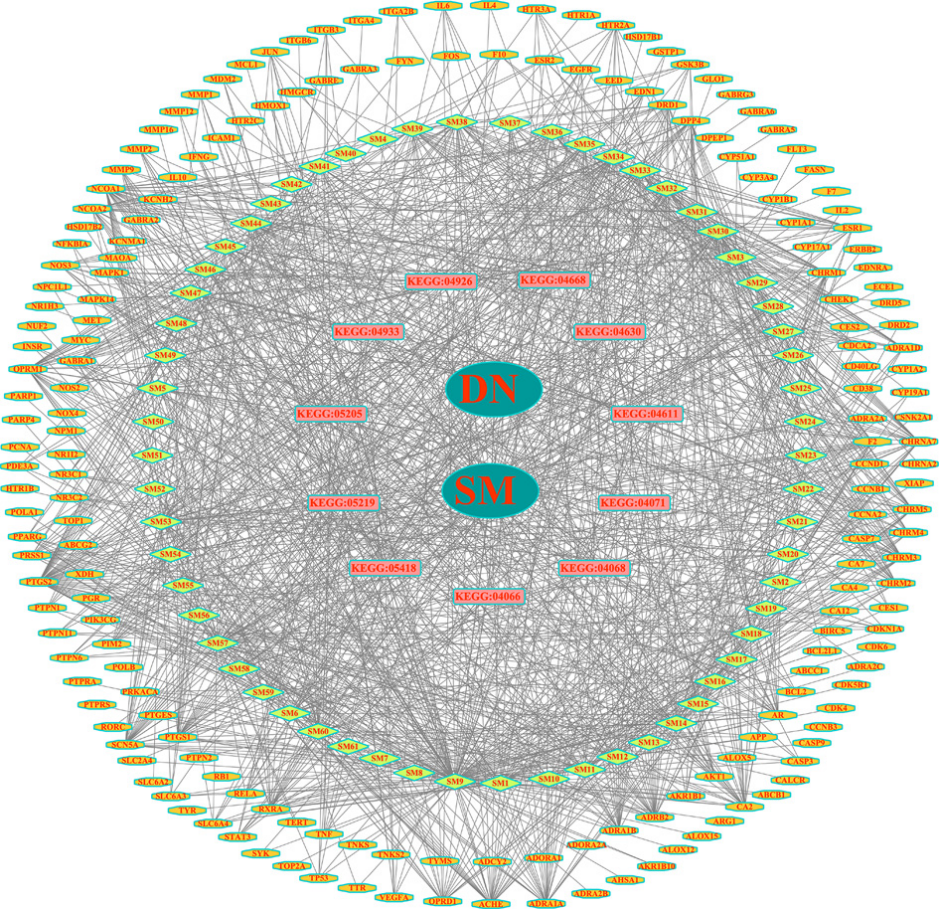
Network structure of ‘SM–component–target pathway–DN’ Ovals represent SM and DN, diamonds represent components, hexagons represent targets, and rectangles represent pathways.

The present study analyzed the mechanisms underlying the therapeutic effect of SM in DN using network pharmacology, by constructing an SM ingredient–target–DN-related pathway network (Figure 8). Our findings revealed that SM exerts pharmacological effects in DN in a multicomponent–multitarget–multipathway manner, including advanced glycation end-products, oxidative stress, inflammatory response, and immune regulation. Our findings offer a reference for further investigation of the mechanism underlying the therapeutic effect of SM in DN.

## Supplementary Material

Supplementary Tables S1-S4

## Data Availability

The datasets used and/or analyzed during the current study are available from the corresponding authors on reasonable request.

## Abbreviations

ADME: absorption, distribution, metabolism, and excretion
AKT1: AKT serine/threonine kinase 1
BP: biological process
CC: cellular component
DL: drug-likeness
DN: diabetic nephropathy
EGFR: epidermal growth factor receptor
ESRD: end-stage kidney disease
GO: Gene Ontology
IL6: interleukin 6
KEGG: Kyoto Encyclopedia of Genes and Genomes
MAPK: mitogen-activated protein kinase
MF: molecular function
OB: oral bioavailability
PPI: protein–protein interaction
SM: *Salvia miltiorrhiza*
STAT3: signal transducer and activator of transcription 3
STRING: Search Tool for the Retrieval of Interacting Genes/Proteins database
TCM: traditional Chinese medicine
TCMSP: TCM Systems Pharmacology
TNF: tumor necrosis factor
TP53: tumor protein p53
